# Consumption experience, choice experience and the endowment effect

**DOI:** 10.1007/s40881-017-0044-z

**Published:** 2017-11-27

**Authors:** Steven J. Humphrey, Luke Lindsay, Chris Starmer

**Affiliations:** 1 School of Business Administration and Economics, University of Osnabrück, Rolandstraße 8, 49069 Osnabrück, Germany; 2 Department of Economics, University of Exeter, Rennes Drive, Exeter, EX4 4PU UK; 3 School of Economics, University of Nottingham, University Park, Nottingham, NG7 2RD UK

**Keywords:** Endowment effect, Experience, Splitting effects, C91, D12

## Abstract

We report experiments investigating how experience influences the endowment effect. Our experiments feature endowments which are bundles of unfamiliar consumption goods. We examine how a subject’s willingness to swap items from their endowment is influenced by prior experiences of tasting the goods in question and by prior experiences of choosing between them. We do not find a statistically significant endowment effect in our baseline treatment and, because of this, we are unable to test for an effect of consumption experience. We do find an endowment effect when the endowment is acquired in two instalments and, in this setting, we find some evidence that choice experience increases trading. In a follow-up experiment, we find evidence that the absence of an endowment effect in our baseline treatment is due to subjects being more willing to swap when they do not have to give up the last unit of their endowment.

**Electronic supplementary material:**

The online version of this article (10.1007/s40881-017-0044-z) contains supplementary material, which is available to authorized users.

## Introduction

We present new experiments studying determinants of the ‘endowment effect’ (see Thaler 1980; Kahneman et al. 1990, 1991). We use the term to refer to a behavioural tendency for people to value goods more highly when they own them, relative to when they do not. The simplest experiments demonstrating endowment effects involve variants of Knetsch’s (1989) ‘swapping task’. When subjects are randomly endowed with one of two items and then given the opportunity to swap their endowment for the other item, the majority choose not to swap. This is an anomaly relative to standard preference theory which predicts a trading rate of 50%. Studies which show that willingness-to-accept valuations for goods are often significantly higher than willingness-to-pay valuations have also been interpreted as evidence of endowment effects.^1^ Endowment effects have been found for both a wide range of goods (lotteries, mugs, candy, toys, memorabilia, stationery, food and drink) and subjects (children, undergraduates, and non-student adults).

Although the endowment effect has been widely observed, evidence suggests it may be eroded by certain kinds of experience. Much of the existing evidence relates to the influence of various forms of *market* experience. For example, when valuations are elicited repeatedly in experimental markets, the gap between willingness-to-accept and willingness-to-pay usually decays (e.g. Shogren et al. 2001; Loomes et al. 2003). List (2003, 2004) reports that experienced traders in a naturally occurring market show no endowment effect. Engelmann and Hollard (2010) find that subjects who have previously been ‘forced’ to trade exhibit no endowment effect in subsequent swapping tasks.

We focus on experiences that are separable from market participation. We investigate two types of experience that arise commonly in daily life and which, we conjectured, might influence the extent of an endowment effect: these are experiences arising, respectively, from *consuming* and from *choosing* goods. In the next section, we discuss background theory. Section 3 sets out our experimental design, Sect. 4 presents results, Sect. 5 reports the results of a follow-up experiment and Sect. 6 concludes.

## Background theory

We draw on a theory proposed by Loomes et al. (2009) (henceforth LOS) to motivate our experiment. LOS propose a model of consumer choice which predicts the endowment effect as a consequence of two factors: individuals are uncertain about the utility an alternative will deliver and they are loss averse. In LOS, preferences are defined over consumption bundles. Each bundle *x* is a set of consumption characteristics represented as an act (Savage 1954), which associates a specific utility *U*_*s*_(*x*) with each element *s*, of a state space *S*. The state space represents ‘taste uncertainty’ which can arise from *extrinsic* or *intrinsic* sources. For example, when in a restaurant and considering the act “order fish”, *extrinsic* uncertainty may exist in relation to whether the fish will be cooked well or not, while *intrinsic* uncertainty might reflect an individual’s lack of clarity about their own preference (e.g. not being sure whether one is *in the mood* for fish).^2^

How LOS explain the endowment effect can be illustrated with a simple example. Imagine a choice between two acts *x* and *y*, defined over two equally probable states of the world, *s*_1_ and *s*_2_. Act *x* yields utility of 1 in *s*_1_ and 0 in *s*_2_. Act *y* yields utility of 0 in *s*_1_ and 1 in *s*_2_. Assume that *y* is the status quo and consider the option of switching to *x*. Under *s*_1_, switching would provide a *gain* in utility of 1 and under *s*_2_ switching results in a *loss* in utility of 1. When faced with this uncertainty, a consumer who is loss-averse in utility would maintain the status quo (regardless of whether this was *x* or *y*); hence, there is an endowment effect.

### The taste uncertainty hypothesis

A distinctive property of the LOS model is that “the strength of status quo effects is positively related to the extent of taste uncertainty” (LOS, p. 132). The intuition follows from the previous example. In the absence of taste uncertainty, either *s*_1_ or *s*_2_ occurs for sure. The individual then has a strict preference ranking of the two acts which is independent of the status quo.^3^ It follows that experiences which reduce taste uncertainty can, other things equal, weaken the endowment effect. We call this the *taste uncertainty hypothesis* and in Sect. 3 we present an experiment designed to test it.

### The choice experience hypothesis

Our experiment was also designed to test the conjecture that prior experiences of making choices between specific goods may weaken a subsequent endowment effect, relative to those goods. We call this conjecture the *choice experience hypothesis*. While we do not know of a current theory which specifically predicts this, more than one plausible psychological mechanism might work in this direction.

One interpretation of the endowment effect is that prior endowments create *biases*, causing stated preferences to deviate from underlying preferences (Samuelson and Zeckhauser 1988 and Plott 1996 offer interpretations in this spirit). Given a bias interpretation, it is possible that prior experiences of choosing between a pair of goods, pre-endowment, could diminish any subsequent endowment effect. Imagine, for instance, an individual who accumulated multiple experiences via considering the ranking of a pair of alternatives from different initial endowment positions (e.g. owning one, owning the other, or owning neither). It seems plausible to suppose that such experiences might provide an individual with perspective on their own preferences which renders them less susceptible to bias.

In addition to the possible debiasing role of prior choice, other possible mechanisms might cause prior choice to reduce an endowment effect. For example, as explained above, in the LOS model the strength of the endowment effect may be positively related to the degree of intrinsic uncertainty associated with a choice. So, if making a choice between two goods reduces vacillation in subsequent decisions over the same pair of goods (perhaps, for instance, because the individual has some preference for consistency), that would provide another conduit for operation of the choice experience hypothesis, consistent with the LOS model.

## The experiment

**Fig. 1:**
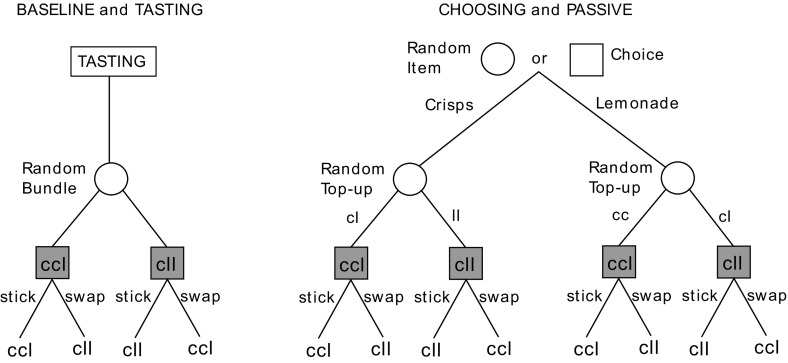
The treatments

We test the *taste uncertainty hypothesis* by investigating whether consumption experience, in an environment where subjects are uncertain about how much they will enjoy available alternatives, reduces the endowment effect. We do this by comparing behaviour in two treatments which we label BASELINE and TASTING. These two treatments are represented in the left-hand tree in Fig. 1.

Our BASELINE treatment was a variant of Knetsch’s (1989) classic swapping task, where subjects were randomly allocated one of two possible endowments and then given the opportunity to either stick or swap. On Fig. 1, the treatment began at the circular node denoting the random allocation. The swap decision is denoted by the shaded square nodes. Relative to the classic task, our BASELINE treatment has two distinguishing features. The first is that the goods which comprised the endowments were consumption goods selected in the expectation that subjects *would be unsure how much they might like them*. The two goods were premium organic vegetable crisps and handmade organic lemonade (for full details see the supplementary materials). The goods were supplied by specialist wholesalers and had similar retail prices of approximately £2. The limited availability and premium nature of the goods meant that subjects were unlikely to have tasted them before. The second distinctive feature of our BASELINE treatment was that each endowment was a *bundle* of goods rather than a single object: each subject was randomly endowed with either a ‘crisps-rich’ bundle consisting of two packets of crisps and a single bottle of lemonade (which we denote ccl) or a ‘lemonade-rich’ bundle consisting of a single packet of crisps and two bottles of lemonade (denoted cll). The rationale for using bundles is explained below.

The TASTING treatment was identical to the BASELINE treatment except that each subject consumed a small amount of the two goods before being endowed with their bundle. Starting at the top of Fig. 1, the treatment began with a subject tasting samples of each good. The experimenter then allocated one of the two endowments at random. This procedure placed each subject at one of the shaded decision nodes where they were faced with a choice between sticking with their allocated endowment or swapping it for the other one. This decision determined a final allocation which was theirs to keep and take from the experiment.

Under conventional preference theory in which endowments play no role, even if tasting changes a subject’s ranking of the bundles, each subject has a 50% chance of not being endowed with their most preferred bundle. Hence, we should expect a 50% chance of swapping in both the BASELINE and TASTING treatments.

Given the assumptions that (1) the goods used in our experiment are ones for which individuals would have taste uncertainty and that (2) consuming small quantities of these goods would reduce taste uncertainty, the comparison of behaviour in the BASELINE and TASTING treatments provides a simple test of the hypothesis that taste uncertainty contributes to the endowment effect. On that hypothesis, we should expect the endowment effect to be relatively weaker in the TASTING treatment.

Our second main objective was to test the *choice experience hypothesis*. Our test involves a comparison of two treatments which we label CHOOSING and PASSIVE. These treatments employ a novel variant of the swapping task that involves *sequences* of choices. It facilitates a test of the choice experience hypothesis by requiring subjects to choose between the two goods en route to their endowment while, nevertheless, ensuring that they receive a random endowment of goods before facing the swap decision.

The PASSIVE and CHOOSING treatments are described by the right-hand tree in Fig. 1. The only difference between them is what happened at the first node at the top of the figure. In CHOOSING, the first node was a *decision node* at which subjects chose either one packet of crisps or one bottle of lemonade, which they were then physically given. A random device then determined a ‘top-up’ to their endowment, so that with equal probability they would find themselves with either a crisps-rich or a lemonade-rich bundle.^4^ This placed the subject at one of the four shaded choice nodes in the lower part of Fig. 1. Subjects then chose whether to stick with their endowment or swap it for the other bundle. The PASSIVE treatment was exactly the same, except that the initial component of the endowment (at the first node) was determined by chance rather than by the subject’s own choice.

This “top-up” method ensures that the bundle held at the point of the swap decision is randomly determined in both the PASSIVE and CHOOSING treatments, and note that it is independent of the initial choice in the latter. Hence, the prediction of a 50% swap rate based on standard theory applies to both. Comparing behaviour between these treatments provides a test of the choice experience hypothesis. On that hypothesis, we should expect more swaps in the CHOOSING treatment than in the PASSIVE treatment.

To test our hypotheses, 210 subjects recruited at the University of Nottingham, were randomly assigned to our four treatments. Each treatment had around 50 subjects. For an effect size with Cohen’s *h* = 0.5 (approximately the difference between a 0.25 and a 0.5 swap rate) and a 0.05 significance level, the power is 80% for within treatment tests (i.e. tests for an endowment effect) and 70% for between treatment tests (i.e. tests for differences in swap rates). Following the swap decision, each subject completed a questionnaire. This provides information on individual characteristics which we exploit in the analysis of Sect. 4. Full details of the goods, the tasks and the scripts followed by the experimenters are described in the supplementary materials.

## Results

Table 1 reports swap rates by treatment. The *Endowments* column shows the number of subjects initially endowed with each of the two bundles. The *Swaps* column reports the total number of swaps and (in parentheses) the number of swaps in each possible direction: swapping crisps for lemonade (c → l) or lemonade for crisps (c ← l). The *Swap rate* is the proportion of subjects who swapped. The final column reports *p*-values for Boschloo tests of the null hypothesis (based on standard preference theory) that the final allocation is independent of endowment (i.e. there is 50% swapping rate), against the alternative hypothesis that there is an endowment effect (i.e. the swap rate is less than 50%).^5^

**Table 1:** Endowments and trading by treatment

Treatment	*N*	Endowments (ccl, cll)	Swaps total (c → l, c ← l)	Swap rate	*p* value
BASELINE	50	(25, 25)	21 (10, 11)	0.42	0.1611
TASTING	56	(27, 29)	26 (16, 10)	0.46	0.3460
Total	106	(52, 54)	47 (26, 21)	0.44	0.1394
PASSIVE	52	(26, 26)	12 (6, 6)	0.23	0.0001
CHOOSING	52	(22, 30)	18 (8, 10)	0.35	0.0177
Total	104	(48, 56)	30 (14, 16)	0.29	0.0000
All	210	(100, 110)	77 (40, 37)	0.37	0.0001

We comment first on the results for the BASELINE and TASTING treatments. While the swap rates for these treatments have the expected pattern with BASELINE < TASTING < 0.5, neither has a statistically significant endowment effect. The absence of an endowment effect in the BASELINE treatment is noteworthy, and we examine this further in Sect. 5. Its absence, however, means that we cannot conduct a meaningful test of the taste experience hypothesis (which would require us to look for a reduction of the endowment effect in TASTING relative to BASELINE).

We now test the choice experience hypothesis by comparing behaviour between the PASSIVE and CHOOSING treatments. There is a significant endowment effect in the PASSIVE treatment (the trading rate is only 0.23) and the experience of choosing weakens it. In line with the choice experience hypothesis, the trading rate rises to 0.35 for the CHOOSING treatment where subjects are approximately 50% more likely to trade. This treatment difference just fails to reach significance at the 10% level (*p* = 0.1023, Boschloo test with one-sided alternative hypothesis) though we do also find a (weakly) significant effect of choice experience in the individual-level analysis below (see analysis of Table 2).

**Table 2:** Logit analysis of swap decisions

	(1)	(2)	(3)	(4)	(5)
Constant	− 0.55*** (0.14)	− 1.09 (1.82)	− 0.47 (0.32)	− 1.49 (1.86)	− 1.55 (1.91)
Female		− 0.33 (0.29)		− 0.37 (0.30)	− 0.37 (0.30)
Age		0.05 (0.09)		0.09 (0.09)	0.09 (0.09)
Loss aversion		− 0.88* (0.51)		− 0.90* (0.52)	− 0.93* (0.52)
Endowed crisps		0.36 (0.29)	0.29 (0.29)	0.34 (0.30)	0.37 (0.30)
TASTING			0.19 (0.39)		0.24 (0.40)
PASSIVE			− 0.89** (0.44)		− 0.94** (0.44)
CHOOSING			− 0.29 (0.41)		− 0.34 (0.42)
Two-step				− 0.75** (0.30)	
*n*	210	210	210	210	210

The dependent variable is 1 if the subject traded, 0 otherwise. Female is 1 if the subject was female, 0 otherwise. Age is measured in years. Loss aversion ranges from 0 (least averse) to 1 (most averse). Endowed crisps is 1 if the subject received a crisps-rich endowment, 0 otherwise. The next three variables are dummies to identify treatments. Two-step is 1 for treatments where endowment is received in instalments. Estimates are logit coefficients (standard errors in parentheses)

*Denotes significance at the *p* < 0.1 level; ** at the *p* < 0.05 level; *** at the *p* < 0.001 level

An unanticipated feature of our results is the difference between the treatments in which the acquisition of endowments occurred in two steps (PASSIVE, CHOOSING), rather than one step (BASELINE, TASTING). While 44% of subjects swapped in the one-step treatments, only 29% of subjects did so in two-step treatments (*p* = 0.0190, Boschloo test with two-sided alternative hypothesis). Comparing the BASELINE and PASSIVE treatments, which control for the experiences of choosing between and tasting the goods, respectively 42% and 23% of subjects swapped their endowment (*p* = 0.0461, Boschloo test, two-sided alternative hypothesis). These tests provide evidence that acquiring an endowment in stages strengthens the endowment effect. We think this is an intriguing discovery and briefly discuss its interpretation and potential significance in Sect. 6.

We supplement the analysis of treatment effects by using logit regression (following List 2003) to model the probability that a subject swaps, taking account of individual characteristics. Observations from all treatments are pooled. This provides a clear overall view of treatment effects within the models we estimate (specifically models 3 and 5) and increases the statistical power of the tests. Across different specifications, as independent variables, we included a dummy for the treatment, the individual experiences, plus a set of individual-level characteristics elicited in the post-decision questionnaire, including age and gender. We also included a measure of individual-level loss aversion constructed by ranking subjects’ from least to most loss averse based on their responses to a series of hypothetical tasks (see supplementary materials). The results are reported in Table 2.

Model 1, which includes only a constant, provides a simple econometric test for the presence of an endowment effect. The highly significant negative coefficient confirms the presence of an endowment effect in our data.

In all three models that include individual-level characteristics (models 2, 4 and 5), the coefficient for measured loss aversion is negative (other characteristics are never significant). Tests of the null hypothesis that the swap rate is independent of loss aversion are rejected at the 5% level (model 2, *p* = 0.0436; model 4, *p* = 0.0424; model 5, *p* = 0.0369). Hence, in these data, more loss averse individuals were less likely to trade. While this result supports theories, including LOS, which invoke loss aversion to explain the endowment effect, we note that we do not replicate this association in the follow-up study reported in Sect. 5.

Models 3 and 5 provide evidence that the experience of choosing part of the endowment increases the trading rate (and reduces the endowment effect). Tests of the null hypothesis that the trading rates in the PASSIVE and CHOOSING treatments are equal are rejected at the 10% level in favour of the alternative hypothesis that the trading rate is *higher* in the CHOOSING treatment (model 3, *p* = 0.0898; model 5, *p* = 0.0894).

Finally, this analysis confirms that acquiring endowments in two steps decreases the trading rate (increases the endowment effect). This is evidenced by the significant negative coefficients on PASSIVE in models 3 and 5 and by the significant coefficient for ‘Two-step’ in model 4.

## Follow-up experiment

A notable feature of the above results is our failure to find a significant endowment effect in our BASELINE treatment, which is closest to the classic swapping task of Knetsch (1989). In this section, we report a simple follow-up experiment designed to diagnose that result.^6^

The most obvious difference between our experiment and other comparable studies which have found an endowment effect is that we endowed subjects with bundles of goods rather than single items. For instance, in Knetsch’s study, subjects who swapped their endowment were giving up their *only* mug or *only* chocolate bar; whereas, in our experiment, subjects were giving up only one of two packets of crisps or one of two bottles of lemonade. It is possible that loss aversion may be more acute in situations where one would be giving up the last unit of a good. However, since there is some evidence that the extent of an endowment effect may depend on the nature of the goods being traded (e.g. Isoni et al. 2011), another possibility is that the absence of an endowment effect in our BASELINE treatment is explained by features of the relatively unfamiliar goods which subjects encountered in our experiment. Our follow-up experiment discriminates between these possibilities.

The follow-up experiment had two treatments. The first replicated our original BASELINE treatment; the second was the same except that endowments were *single items* of one or other of two unfamiliar goods (as in our original experiment, the two goods were either a bottle of an unfamiliar brand of lemonade or a packet of an unfamiliar brand of vegetable crisps). The experiments were conducted at the University of Exeter. A total of 184 subjects participated, with 92 in each treatment. Within each treatment, half of the subjects had each endowment. We used more subjects per treatment in the follow-up experiment to increase statistical power. If the effect size has Cohen’s *h* = 0.4 (approximately the difference between a 0.3 and a 0.5 swap rate) and the significance level is 0.05, 92 subjects per treatment gives 99% power for within treatment tests for an endowment effect and 77% power for between treatment tests for differences in swap rates. Testing for smaller effect sizes requires considerably more observations.

In the baseline replication (with bundles), there were 39 swaps (28 to lemonade, 11 to crisps). This replicates the BASELINE finding reported in Sect. 4: the swap rate (0.42 in the follow-up) is very similar and there is no statistically significant endowment effect. In the single items treatment, there were 33 swaps (31 to lemonade, 2 to crisps). The swap rate was 0.36, giving a statistically significant endowment effect (*p* = 0.0003, Boschloo test with one-sided alternative hypothesis). These results clearly implicate the use of bundles (as opposed to unfamiliar goods) as the culprit for eliminating the endowment effect in our original BASELINE treatment.^7^

## Discussion and conclusion

Our initial design was set up to test two hypotheses partly motivated by existing theory and evidence: the *taste uncertainty hypothesis* and the *choice experience hypothesis*.

We find some evidence that the experience of having made a straight choice between a pair of goods *reduces* the endowment effect observed in a later swap task involving those same goods. While we have found only modest support for this effect, there is a case for further investigation because the operation of it appears to cohere with emerging theory and evidence. From a theoretical viewpoint, the choice experience hypothesis can be interpreted as an implication of the LOS model. We view this theory as an attractive putative account of our data because it models mechanisms which may explain not only why the endowment effect occurs, but also why it changes as a consequence of particular types of experience. From an empirical viewpoint, we see a possible parallel with Engelmann and Hollard (2010). They conjectured that endowment effects may be partly caused by individuals having biased assessments of the costs associated with trading (including mental costs associated with bargaining or deciding). To test this hypothesis, their experiment forced some subjects to trade before they encountered swapping tasks and subjects who had traded as a consequence of this ‘therapy’ exhibited no endowment effect. The reduced endowment effect in our CHOOSING treatment may be evidence that exercising choice en route to an endowment had a comparable ‘therapeutic’ role.

We were unable to test the taste uncertainty hypothesis because, counter to our expectations, we found no endowment effect in our BASELINE condition. We suggested that the most likely candidates for explaining this are one or both of two differences between our BASELINE treatment and a classic swaps design. The first candidate is that we used unfamiliar goods and second is that, in order to facilitate our test of the choice experience hypothesis, subjects chose between bundles of goods rather than single items. Our follow-up experiment provides clear evidence that the use of bundles (not the unfamiliarity of the goods) is the factor which most likely suppressed the endowment effect in our BASELINE treatment. Although it was not part of our initial strategy to test the influence of bundles, we view this aspect of our results as identifying a psychologically plausible determinant of the endowment effect. That is, people are less prone to it when they do not have to give up their final unit of an endowed good.

A final intriguing finding is that even though there was no endowment effect for bundles acquired in a single step, it re-emerged for bundles acquired in two steps. This result, while unanticipated, is possibly related to so-called “splitting effects” reported across a broad range of decision contexts (e.g. Starmer and Sugden 1993; Humphrey 1995; Bateman et al. 1997; Weber et al. 1988).^8^ The common feature of splitting effects is a tendency for a good to be more highly valued when re-described so that positive attributes are unpacked into sub-components (e.g. the “high performance” of a car may be unpacked into sub-categories such as “acceleration”, “handling”, etc.). If splitting effects in bundle acquisition do promote endowment effects, the latter may be particularly pronounced in markets where endowments are built up over time. These may range from markets for relatively low value goods (e.g. memorabilia collections) through to economically large investments such as building a home or a business.

## Electronic supplementary material

Below is the link to the electronic supplementary material.

Supplementary material 1 (DOCX 195 kb)

## Footnotes

1 Horowitz and McConnell (2002) review many of these studies; see also Plott and Zeiler (2005) and Isoni et al. (2011) for further discussion of the interpretation of endowment effects.

2 LOS do not presume that an individual is necessarily self-conscious of their intrinsic uncertainty but we think the restaurant choice example provides an everyday illustration of the underlying idea, consistent with their model.

3 Taste uncertainty might explain why endowment effects occur for some goods but not others. For example, Isoni et al. (2011) observe an endowment effect for lotteries (which are intrinsically uncertain) but not mugs.

4 Subjects were not told the distribution of the top-ups, so did not know their choice would have no effect on their second-stage allocation.

5 The Boschloo test was chosen for two reasons. First, it is an exact test. Unlike the *Z*-test of proportions or the Chi squared test, the Boschloo test does not rely on using an approximation of the test statistic’s distribution. Second, unlike Fisher’s exact test, the Boschloo test does not rely on contingency tables having fixed row and column totals. In the experiment, the row totals (number of subjects in a treatment) were fixed, but the column totals (e.g. total trades across treatments) were not.

6 We thank the editor and a referee for suggesting we develop a diagnostic follow-up experiment.

7 In the follow-up experiment, subjects also completed an incentivized loss aversion elicitation task (details are included in the supplementary materials). In contrast to the first experiment, we did not find a statistically significant correlation between measured loss aversion and swapping.

8 Another possibility is that it is related to evidence that the valuation of an endowment is increasing with the duration the endowment has been held (Strahilevitz and Loewenstein 1998). In our experiment, however, the durational difference between one and two-step treatments is very small.
