# Supplementary material for: Consumption experience, choice experience and the endowment effect
Source: J Econ Sci Assoc. 2017 Nov 27;3(2):109–20. doi: 10.1007/s40881-017-0044-z (PMC6956941; doi:10.1007/s40881-017-0044-z)
Supplement: Supplementary file 1 — Supplementary material 1 (DOCX 195 kb) [file 40881_2017_44_MOESM1_ESM.docx]

Supplementary Materials

First set of experiments (conducted in Nottingham)

75cl lemonade and 100g crisps


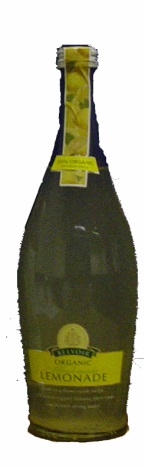

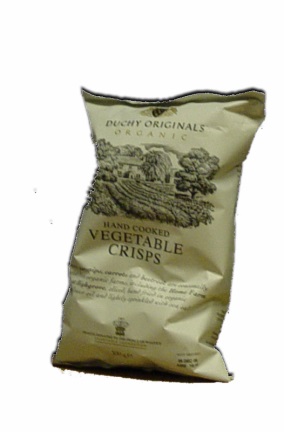


Participant number: ___

Age: __________________________________________ Subject studied: ________________________________

Year of study ___________________________________

Gender: _______________________________________

1. Have you ever bought or sold items on online auctions such as EBay?

No

Yes

1. Have you taken part in CeDEx experiments before?

No

Yes

Participant number: ____

The next 7 tasks are hypothetical because they involve large amounts of money. However, even though you won’t win or lose money in these tasks, please think about them and try to respond to them as if they were for real. Imagine that you are given the choice between playing and rejecting each of the bets. If you play a bet, a coin is flipped. If the coin lands tails up, you lose the amount of money shown in the ‘Tails’ column of the table. If the coin lands heads up, you win an unspecified amount. If you reject a bet, no coin is flipped and you don’t win or lose anything.

For each of the bets, please enter the amount in the win column that is just enough to tempt you to play the bet – that is, the lowest figure for which you would choose to play rather than reject the bet. Please write the amount in pounds in the space provided. For the purposes of this task, imagine that any losses could be paid back over a period of time in the same way that you might payoff a credit card debt and that the interest rate you would pay is 6%.

|  | Tails  LOSE | Heads WIN |
| --- | --- | --- |
| Bet 1 | £100 | ?£........... |
| Bet 2 | £240 | ?£........... |
| Bet 3 | £580 | ?£........... |
| Bet 4 | £1,400 | ?£........... |
| Bet 5 | £3,300 | ?£........... |
| Bet 6 | £7,900 | ?£........... |
| Bet 7 | £19,000 | ?£........... |

Experimenter Scripts

## First Experimenter

The text in **bold** was read out by the experimenter. Some parts of the script differed between treatments. The labels in *italics* indicate which treatments each part of the script applied to.

*All*: The subject was invited to take a seat opposite the experimenter. **This is an experiment looking at how people make decisions. During the experiment you will be asked to make decisions and answer some questions on paper forms.** Depending on which of the four treatments the subject was assigned to, the experiment proceeded as follows.

*Baseline*: **To start with, please could you complete this form.** The experimenter handed the subject the demographic survey and a pen.

*Taster*: **To start with, please could you taste these two items.** The experimenter poured the subject a small cup of lemonade and offered them a small bowl containing some vegetable crisps. The packaging of the two items were in front of the subject so they could examine them if they wished. When the subject had tasted both items, the experimenter handed the subject the demographic survey and a pen, and said **Then could you complete this form**

*Passive*: **To start with, please could you pick an envelope and look inside.** The experimenter offered the subject a basket containing a number of envelopes. The subject picked an envelope and looked inside. It contained a paper token for either one bottle of lemonade or one packet of crisps. Depending on what the token was for, the experimenter handed the subject either a bottle of lemonade or packet of crisps and said **This is yours to keep.** Then the experimenter handed the subject the demographic survey and a pen, and said **Then could you complete this form**

*Chooser*: The experimenter placed a bottle of lemonade and packet of crisps in front of the subject and said **Please could you look at these two items and choose one of them:** **you get to keep the one you choose.** The subject chooses one of the items. Then the experimenter handed the subject the demographic survey and a pen, and said **Then could you complete this form.**

*All*: The experimenter then presented the subject with a basket of envelopes and said **please could you pick an envelope and look inside.** Inside the envelope was a token for several units of the two goods. The experimenter handed the subject the goods specified by the token.

The experimenter then asked the subject to complete the loss aversion survey. Once the subject had completed the survey the experimenter said **The first part of the experiment is complete**. Drawing the subject’s attention to the items they have been given: **These are yours to keep. Please could you take them around the corner and [Name of second experimenter] will complete the second part of the experiment with you.**

## Second Experimenter

The script followed by the experimenter depended on which combination of crisps and lemonade the subject had received in the first part of the experiment.

*Subject has 2 packets of crisps*: **You have two packets of crisps and one bottle of lemonade. If you want, you can swap one of your packets of crisps for a bottle of lemonade.**

*Subject has 2 bottles of lemonade*: **You have two bottles of lemonade and one packet of crisps. If you want, you can swap one of your bottles of lemonade for a packet of crisps.** The experimenter swapped the item if the subject wished to swap, and recorded the decision. Then the experimenter asked the subject to complete a debrief survey and receipt form. Finally the experimenter gave the subject a plastic bag to carry their items and thanked the subject for taking part.


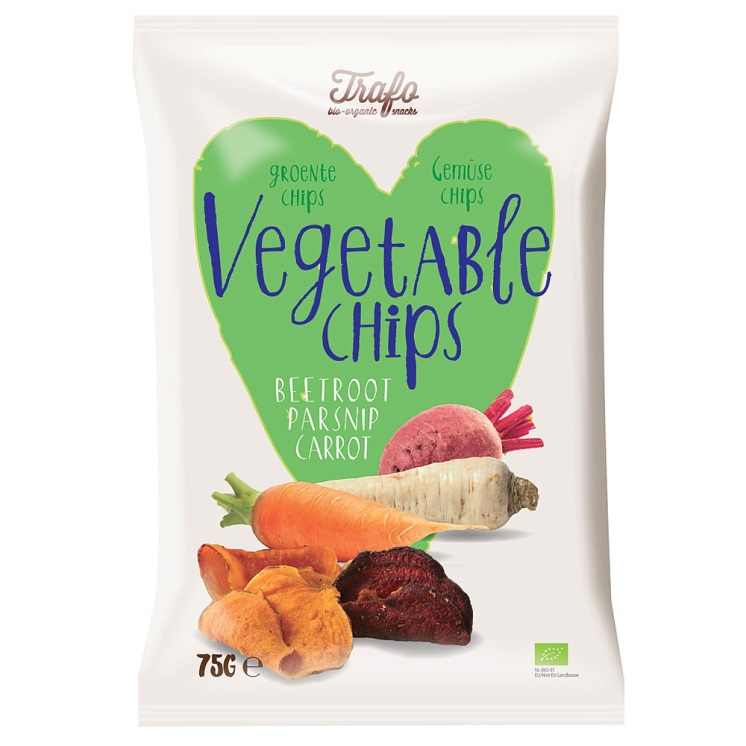

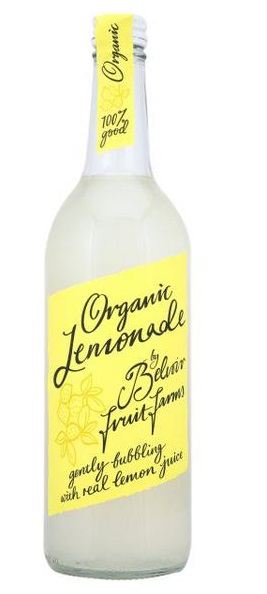
Follow up experiments (conducted in Exeter)

75cl lemonade and 75g crisps.

We ask that you do not communicate with other participants during the experiment.

Please refrain from verbally reacting to events that occur during the experiment.

Please complete the details below and wait for further instructions.

**Participant number:**

**Age:**

**Subject studied:**

**Year of study**

**Gender:**

Tasks 1 - 6

In tasks 1 -6, you are first given £6. You then have to decide whether you would like to play a lottery in which you can win some more money, but might lose some money. If you choose to play this lottery and lose, then the loss will be subtracted from the £6 you have been given. If you prefer, you can choose not to play the lottery, in which case you will simply keep the £6. If, however, you choose to play the lottery and you win, the prize will be added to your £6 and you will keep both.

In these questions, your choice should be recorded by writing a cross in the box underneath whatever you choose to do (“Accept” or “Reject”, i.e. 🗷 ).

One of tasks 1 to 6 will be chosen as your payment-relevant task. This will be done by rolling a dice. The number on the dice will determine which task is chosen. If you accepted the lottery for the chosen task, a coin will be tossed to determine the outcome of the lottery. Any losses will be subtracted from the £6 and any winnings will be added to it. If you rejected the lottery, the coin will not be tossed and nothing will be added to or subtracted from the £6.

Any questions?

| Lottery | Accept | Reject |
| --- | --- | --- |
| #1. If the coin is heads, you lose £1; if the coin is tails, you win £5. | 🞎 | 🞎 |
| #2. If the coin is heads, you lose £2; if the coin is tails, you win £5. | 🞎 | 🞎 |
| #3. If the coin is heads, you lose £3; if the coin is tails, you win £5. | 🞎 | 🞎 |
| #4. If the coin is heads, you lose £4; if the coin is tails, you win £5. | 🞎 | 🞎 |
| #5. If the coin is heads, you lose £5; if the coin is tails, you win £5. | 🞎 | 🞎 |
| #6. If the coin is heads, you lose £6; if the coin is tails, you win £5. | 🞎 | 🞎 |

Experimenter Script

The text in **bold** was read out by the experimenter. Some parts of the script differed between treatments. The labels in *italics* indicate which treatments each part of the script applied to.

Upon entry to the lab, subjects drew a card with a number indicating their seat. At each seat was a consent form and demographics survey.

The experimenter waited for all subjects to be seated.

**This is an experiment looking at how people make decisions. During the experiment you will be asked to make decisions and answer some questions on paper forms. To start with, please could you read through the consent form and sign it if you are happy with it. If you have any questions, please let me know.**

The experimenter waited for all subjects to read through the consent form and sign it.

**If you have signed the consent form, please also complete the other form.**

**I am now going to hand out some items. What you are given is yours to keep.**

The experimenter handed out the item(s) comprising subjects’ endowments, placing the items on the subject’s desk. In each session, subjects were evenly divided between the two sides of the lab. Subjects on one side received a crisps rich endowment and those on the other side a lemonade rich one.

**The next part of the experiment involves making some decisions involving risk. Please read through the form I hand out carefully, and then make your decisions. If you have any questions, please let me know.**

The experimenter handed out the form for the loss aversion elicitation task.

The experimenter waited for all subjects to complete the task.

*Single items:* **This is the final task. What you have in front of you is yours to keep but you have the option to swap it for the item I am about to show you.**

*Single items:* The experimenter walked around the lab showing subjects the alternative item.

*Bundles:* **This is the final task. What you have in front of you is yours to keep but you have the option to swap one of your items.**

The experimenter handed out a simple form on which the subjects were asked to indicate by ticking whether they wanted to keep what they had or make the relevant swap.

Once all subjects had made the swap decision, subjects were called by seat number to be paid individually by the experimenter. Swaps were implemented. A die and coin were used to resolve the loss aversion task individually for each subject, and subjects were paid according to the outcome.
